# Evaluation of the Roche cobas MTB and MTB-RIF/INH Assays in Samples from Germany and Sierra Leone

**DOI:** 10.1128/JCM.02983-20

**Published:** 2021-04-20

**Authors:** Darshaalini Nadarajan, Doris Hillemann, Rashidatu Kamara, Lynda Foray, Ousman S. Conteh, Matthias Merker, Stefan Niemann, Jasmine Lau, Merlin Njoya, Katharina Kranzer, Akos Somoskovi, Florian P. Maurer

**Affiliations:** a National and Supranational Reference Laboratory for Mycobacteria, Research Center Borstel, Leibniz Lung Center, Borstel, Germany; b National Leprosy and Tuberculosis Control Programme (NLTCP), Ministry of Health and Sanitation (MOHS), Freetown, Sierra Leone; c German Center for Infection Research (DZIF), Partner Site Hamburg-Lübeck-Borstel-Riems, Hamburg, Germany; d Molecular and Experimental Mycobacteriology, Research Center Borstel, Borstel, Germany; e Roche Molecular Systems, Pleasanton, California, USA; f London School of Hygiene and Tropical Medicine, Clinical Research Department, London, United Kingdom; g Institute of Medical Microbiology, Virology and Hygiene, University Medical Center Hamburg-Eppendorf, Hamburg, Germany; University of Manitoba

**Keywords:** tuberculosis, molecular diagnostics, PCR, rifampicin, isoniazid, multidrug resistance, sputum

## Abstract

The Roche cobas MTB and MTB-RIF/INH assays allow for detection of Mycobacterium tuberculosis complex (MTBC) nucleic acid and rifampicin (RIF) and isoniazid (INH) resistance-associated mutations in an automated, high-throughput workflow. In this study, we evaluated the performance of these assays, employing samples from settings of low and high tuberculosis (TB) burdens. A total of 325 frozen, leftover respiratory samples collected from treatment-naive patients with presumptive TB in Germany (*n* = 280) and presumptive RIF-resistant TB in Sierra Leone (*n* = 45) were used in this study. cobas MTB results for detection of MTBC DNA from *N*-acetyl-l-cysteine–sodium hydroxide (NALC–NaOH)-treated samples were compared to culture results. Predictions of RIF and INH resistance by the cobas MTB-RIF/INH assay were compared to a composite reference standard (phenotypic drug susceptibility testing and line probe assay). Whole-genome sequencing was used to resolve discordances. The overall sensitivity of cobas MTB for detection of MTBC DNA in culture-positive samples (*n* = 102) was 89.2% (95% confidence interval [CI], 81.7 to 93.9%). The specificity of cobas MTB was 98.6% (95% CI, 96.1 to 99.5%). Sensitivity and specificity for detection of RIF and INH resistance were 88.4% (95% CI, 75.5 to 94.9%) and 97.6% (95% CI, 87.4 to 99.6%) and 76.6% (95% CI, 62.8 to 86.4%) and 100.0% (95% CI, 90.8 to 100.0%), respectively. Discordant results for RIF and INH resistance were mainly due to uncommon mutations in samples from Sierra Leone that were not covered by the cobas MTB-RIF/INH assay. In conclusion, cobas MTB and MTB-RIF/INH assays provide accurate detection of MTBC DNA and resistance-associated mutations in respiratory samples. The influence of regional variations in the prevalence of resistance-conferring mutations requires further investigation.

## Abstract (precis)

The cobas MTB and MTB-RIF/INH assays allow for detection of Mycobacterium tuberculosis complex (MTBC) nucleic acid and rifampicin (RIF) and isoniazid (INH) resistance-associated mutations in an automated, high-throughput workflow. In this study, we evaluated the performance of these assays, employing samples from settings of low and high tuberculosis (TB) burdens.

## INTRODUCTION

In 2019, there were an estimated 1.2 million deaths from tuberculosis (TB) among HIV-negative people and 10 million new TB cases worldwide (1). Approximately 4% of these cases were caused by multidrug-resistant (MDR) strains of Mycobacterium tuberculosis complex (MTBC), which are characterized by resistance to at least the two most effective first-line drugs, isoniazid (INH) and rifampicin (RIF) (1–3). Treatment success rates are lower for MDR-TB than for drug-susceptible TB (57% and 85%, respectively) (1), clearly falling short of the World Health Organization (WHO)-devised End TB Strategy target of a ≥90% treatment success rate by 2025 (4). Additionally, INH- and RIF-monoresistant forms of TB are also associated with poorer patient outcomes (5, 6) and are highly prevalent in some high-burden settings (7–9). For example, in South Africa (2012 to 2014), the prevalence of INH-monoresistant TB was >5% in all provinces (9). The prevalence of new RIF-resistant TB cases (3.4%) almost doubled compared with 2001-2002 data (1.8%), while the prevalence of MDR-TB remained stable (2.8% versus 2.9%) (9).

Consequently, the End TB Strategy has renewed the call for universal drug susceptibility testing to be available for patients with confirmed TB (1, 4). However, conventional phenotypic drug susceptibility testing (pDST) is time-consuming and complex, involving culturing of clinical samples in a biosafety level 3 facility, which is not always available in high-burden settings. In order to appropriately identify and treat MDR- as well as INH- and RIF-monoresistant TB, rapid detection of resistance-conferring mutations for both drugs is required (10). Nucleic acid amplification tests (NAATs) are useful alternative tools for the detection of MTBC and drug resistance markers directly from clinical samples (11). Testing for RIF resistance-associated mutations in the *rpoB* gene and INH resistance-associated mutations in the *katG* gene and *fabG1*-*inhA* promoter region offers the possibility to accelerate identification of drug resistance compared to pDST (2, 11). However, due to the type and frequency of these mutations varying across geographic regions and their disparate impacts on phenotypic resistance profiles, it is important to validate the clinical application of NAATs in various settings (9, 12–14).

The Roche cobas MTB and MTB-RIF/INH assays offer a high-throughput NAAT platform for direct detection of MTBC DNA, and RIF and INH resistance-associated mutations (15) from inactivated human respiratory samples, including raw and processed sputum and bronchoalveolar lavage (BAL) fluid. MTBC DNA-positive samples are subsequently tested for resistance-associated mutations, which allows for diagnosis of RIF or INH monoresistance as well as MDR-TB. Limited data are available for the performance of the cobas MTB and MTB-RIF/INH assays compared to culture and other commercially available NAATs (16, 17).

This study aimed to evaluate the sensitivity and specificity of the cobas MTB and MTB-RIF/INH system in a reference laboratory which receives samples from settings of low (Germany; notification rate, 5.8/100,000 population) and high (Sierra Leone; notification rate, 295/100,000) TB burdens (18).

## MATERIALS AND METHODS

### Study design and ethics.

This was a single-center, retrospective evaluation using remnant samples submitted to the German National Reference Center for Mycobacteria for testing, until the target number of samples needed to meet the study objectives was obtained (60 MTBC culture-positive and 150 MTBC culture-negative samples). All samples were anonymized prior to inclusion. The study was conducted in compliance with the International Conference on Harmonisation Good Clinical Practice Guideline and local regulations. The study protocol was approved by the relevant institutional review board prior to initiation.

### Samples.

Remnant frozen *N*-acetyl-l-cysteine–sodium hydroxide (NALC–NaOH)-treated sputum samples and BAL fluid sediments of more than 500 μl obtained from individuals suspected of having TB were used in this study. Samples from Sierra Leone that locally screened MTBC positive and RIF resistant by Xpert MTB/RIF (Cepheid, Sunnyvale, CA) were sent to Germany for confirmation by culture, pDST, and molecular DST (mDST) as required. All samples were taken from adults aged >18 years within 2 days of patients starting TB treatment. Samples from patients who were already on treatment were excluded. Cross-reactivity with nontuberculous mycobacteria (NTM) was explored using remnants of 18 microscopically positive sputa obtained in Germany that grew Mycobacterium avium complex, Mycobacterium kansasii, or Mycobacterium abscessus.

### Testing.

Testing was performed at the National Reference Center for Mycobacteria in Borstel, Germany, on a cobas 6800 system using the cobas MTB assay with the cobas MTB positive control, buffer negative control, and microbial inactivation solution (Roche, Rotkreuz, Switzerland). All cobas MTB PCR-positive samples were subsequently investigated for RIF and INH resistance using the cobas MTB-RIF/INH assay. Analytical data obtained during routine diagnostic workups were collected from the electronic laboratory management system. These data included results for fluorescence smear microscopy, growth on solid (one Löwenstein-Jensen and one Stonebrink slant per sample) and liquid (Bactec MGIT 960; Becton, Dickinson, Franklin Lakes, NJ) culture media, pDST results for INH and RIF at the WHO-recommended critical concentrations (CC) of 0.1 mg/liter and 1.0 mg/liter, respectively (19), and results for the GenoType MTBDR*plus* (Hain Lifescience, Nehren, Germany) line probe assay (LPA) where applicable. Results of the Xpert MTB/RIF assay were available for a subset of 128 samples. Selected samples, in particular the discordant samples, were also tested by the Xpert MTB/RIF Ultra assay (Cepheid, Sunnyvale, CA).

### Whole-genome sequencing.

Whole-genome sequencing (WGS) was performed with Illumina technology employing the NextSeq500 and Nextera XT DNA library preparations according to the manufacturer’s instructions (Illumina, San Diego, CA), and the MTBseq pipeline was used for data analysis, as described previously (20). Briefly, raw reads were mapped to the M. tuberculosis H37Rv genome (GenBank accession number NC_000962.3), and variants (single nucleotide polymorphisms and small insertions and deletions) with a minimum coverage of four reads in both forward and reverse orientations, at least four reads calling the variant with a Phred score of ≥20, and a minimum variant frequency of 75% were extracted.

Genes and upstream regions implicated in resistance to INH (*katG*, *fabG1*, *inhA*, *inbR*, *mshA*, *mmaA3*, *mshB*, *sigI*, *ndh*, *furA*, *mshC*, *kasA*, *mymA*, *nudC*, *nat*, and *Rv3083*) and RIF (*rpoB*) were investigated for resistance-associated mutations and interpretation of discordant results. The absence of sequencing reads in the aforementioned genes was reported as large unspecified deletions. Previously identified mutations in these genes that are not correlated with resistance but rather reflect phylogenetic variation were not considered possible resistance determinants (21).

### Analyses.

cobas MTB results were compared to growth of MTBC bacteria in solid or liquid medium as a reference. Smear-negative and smear-positive samples were analyzed separately. cobas MTB data were compared to Xpert MTB/RIF or Xpert MTB/RIF Ultra results where available. For prediction of resistance to INH or RIF, cobas MTB-INH/RIF results were compared to a composite reference standard (CRS) comprising routine pDST (MGIT 960) and mDST by LPA. Samples were flagged resistant as per the CRS if growth occurred at the CC for INH or RIF or in case of detection of a “borderline” mutation associated with elevated RIF MICs below the CC. WGS was used to resolve discordances between the cobas MTB-RIF/INH and CRS results. Statistical analyses were performed using SAS/STAT software.

## RESULTS

### Samples.

**FIG 1:**
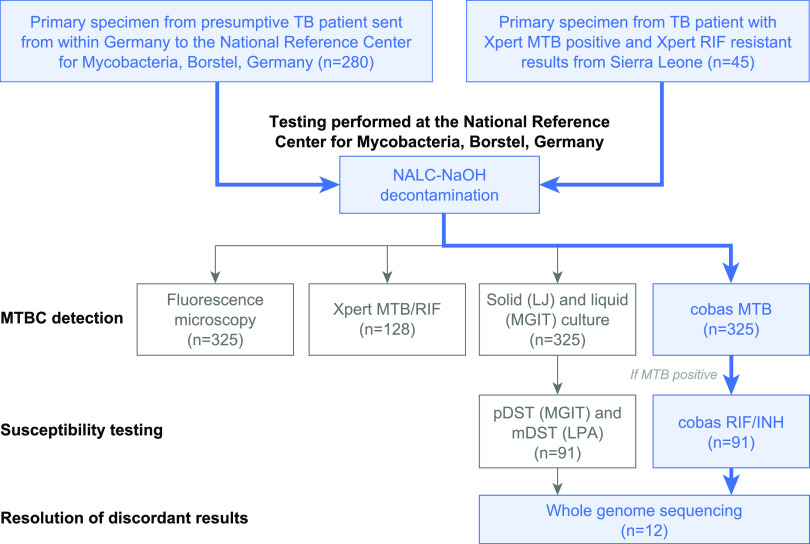
Sample flow and analytical procedures. TB, tuberculosis; NALC–NaOH, *N*-acetyl-l-cysteine–sodium hydroxide; LJ, Löwenstein-Jensen medium; MGIT, mycobacterial growth indicator tube; pDST, phenotypic drug susceptibility testing; mDST, molecular drug susceptibility testing; LPA, line probe assay; RIF, rifampicin; INH, isoniazid.

In total, 325 sputum samples from treatment-naive patients with suspected TB from Germany (*n* = 280 [86.0%]) and Sierra Leone (*n* = 45 [14.0%]) were investigated (1 sample per patient [Fig. 1]). Of patients from Germany, 63.6% were male (*n* = 178), 28.2% were female (*n* = 79), and the sex of 8.2% (*n* = 23) was unknown, with a median age for all patients of 52 years (interquartile range [IQR], 33 to 70). Of patients from Sierra Leone, 71.1% (*n* = 32) were male and 28.9% (*n* = 13) were female, with a median age of 30 years (IQR, 21 to 42).

### Performance of the cobas MTB assay.

The overall sensitivity of the cobas MTB assay for detection of MTBC in culture-positive samples was 89.2% (95% confidence interval [CI], 81.7 to 93.9%) (Table 1). As expected, for smear-positive, culture-positive samples, cobas MTB demonstrated a higher sensitivity (98.7%; 95% CI, 92.8 to 99.8%) than for smear-negative, culture-positive samples (63.0%; 95% CI, 44.2 to 78.5% [Table 1]).

**TABLE 1:** Diagnostic performance of the cobas MTB assay for identification of Mycobacterium tuberculosis complex nucleic acid from primary samples^*a*^

Sample source	No. of samples/total (% [95% CI])			
	Sensitivity			Specificity
	Smear-positive	Smear-negative	Pooled	
Germany	32/33 (97.0 [84.7, 99.5])	14/24 (58.3 [38.8, 75.5])	46/57 (80.7 [68.7, 88.9])	219/222^*b*^ (98.6 [96.1, 99.5])
Sierra Leone	42/42 (100.0 [91.6, 100.0])	3/3 (100.0 [43.9, 100.0])	45/45 (100.0 [92.1, 100.0])	NA^*c*^
All samples	74/75 (98.7 [92.8, 99.8])	17/27 (63.0 [44.2, 78.5])	91/102 (89.2 [81.7, 93.9])	219/222 (98.6 [96.1, 99.5])

a Culture was used as a reference standard. CI, confidence interval.

b A total of 18/222 (8.1%) of the investigated Mycobacterium tuberculosis complex (MTBC)-negative samples grew nontuberculous mycobacteria (Mycobacterium avium complex, *n* = 9; Mycobacterium kansasii, *n* = 6; and Mycobacterium abscessus, *n* = 3).

c Only MTBC culture-positive samples from Sierra Leone were analyzed. NA, not applicable.

With regard to the geographic origin of the samples, the cobas MTB assay correctly detected TB in all of the culture-positive samples from Sierra Leone, which were mostly smear-positive (42/45 samples [93.3%] [Table 1]). Among the 57 MTBC culture-positive samples from Germany, 33 (57.9%) were smear-positive. Consequently, the sensitivity of the cobas MTB assay for detection of MTBC DNA was lower in this sample subset (80.7%; 95% CI, 68.7 to 88.9% [Table 1]).

Since all samples from Sierra Leone were MTBC culture-positive, specificity was investigated based on samples from Germany alone (98.6%; 95% CI, 96.1 to 99.5%). In total, 3/222 MTBC culture-negative samples were classified as positive by the cobas MTB assay. Of those, 2/3 samples were both smear- and culture-negative and 1 grew Mycobacterium chimaera but not MTBC. With regard to the latter, repetition of cobas MTB from the positive MGIT 960 culture gave a negative result; no leftover primary material was available for repeat testing. Information on a history of TB that could explain the positive PCR results was not available.

Cross-reactivity with nontuberculous mycobacteria (NTM) was investigated using 18 microscopically positive sputa that grew M. avium complex, M. kansasii, or M. abscessus (Table 1). cobas MTB was negative in 17/18 NTM culture-positive samples (94.4%; 95% CI, 72.7 to 99.9%), with the one falsely positive cobas MTB result corresponding to a sample that grew *M*. *chimaera* as outlined above.

A comparative analysis between cobas MTB and Xpert MTB/RIF based on 128 samples (smear-positive/MTBC culture-positive, *n* = 60; smear-negative/MTBC culture-positive, *n* = 15; smear-positive/MTBC culture-negative but M. kansasii culture-positive, *n* = 1; and smear-negative/MTBC culture-negative, *n* = 52) showed 99.2% (95% CI, 95.7 to 99.9%) overall agreement for detection of MTBC DNA (see Table S1 in the supplemental material).

### Detection of rifampicin and isoniazid resistance.

Both RIF and INH resistances as per the CRS were more frequent among samples from Sierra Leone than among samples from Germany and more frequent in smear-positive than in smear-negative samples (Table S2). Overall, cobas MTB-RIF/INH demonstrated a sensitivity of 88.4% (95% CI, 75.5 to 94.9%; 38/43 samples) for detection of RIF resistance and a sensitivity of 76.6% (95% CI, 62.8 to 86.4%; 36/47 samples) for detection of INH resistance. Specificities were 97.6% (95% CI, 87.4 to 99.6%; 40/41 samples) for RIF and 100.0% (95% CI, 90.8 to 100.0%; 38/38 samples) for INH (Table 2).

**TABLE 2:** Identification of rifampicin and isoniazid resistance-associated mutations from Mycobacterium tuberculosis complex DNA-positive samples by the cobas MTB-RIF/INH assay^*a*^

Drug	No. of samples/total (% [95% CI])			
	Sensitivity			Specificity
	Smear-positive	Smear-negative	Pooled	
Rifampicin	36/40 (90.0 [76.9, 96.0])	2/3 (66.7 [20.8, 93.9])	38/43 (88.4 [75.5, 94.9])	40/41 (97.6 [87.4, 99.6])
Isoniazid	32/42 (76.2 [61.5, 86.5])	4/5 (80.0 [37.6, 96.4])	36/47 (76.6 [62.8, 86.4])	38/38 (100.0 [90.8, 100.0])

a A combination of phenotypic and molecular (line probe assays) drug susceptibility testing performed from cultures was used as a composite reference standard. CI, confidence interval.

MTBC isolates that displayed either INH (*n* = 9) or RIF (*n* = 5) resistance by the CRS but were not identified by cobas MTB-RIF/INH (15.4% of all results generated by cobas MTB-RIF/INH) were further analyzed using WGS (Table 3). For INH, all samples with discordant results originated from Sierra Leone. Resistance was associated with uncommon mutations in *katG* such as M105I, W397C, or deletion at 792G (del792G), mostly in combination with mutations in other genes associated with INH resistance, such as *fabG1*, *mshB*, and *nudC* (Table 3). For RIF, three discordant samples originated from Sierra Leone and two from Germany. Of the five discordant results, two had entire codon deletions in *rpoB* (at position 517 or 518) and two had point mutations resulting in *rpoB* S531F or *rpoB* Q513E (Table 3). The MTBC isolate grown from the fifth sample had the commonly observed S531L mutation in *rpoB*. Repeat testing was not possible due to the limited volume of the primary sample. Repeat testing by cobas MTB-RIF/INH from the corresponding MTBC culture isolate correctly identified the isolate as RIF resistant. Of note, the S531L mutation was correctly detected in 21 unrelated samples. Excluding this sample, the sensitivity of cobas MTB-RIF/INH for determining RIF resistance would have increased to 90.5% (*n* = 38/42).

**TABLE 3:** Resolution of discordant results for prediction of resistance to isoniazid and rifampicin^*a*^

Drug	Sample	Country	Cobas MTB RIF or INH target^*b*^	CRS		Xpert MTB/RIF Ultra	WGS^*c*^	Interpretation
				pDST	LPA			
INH	18000119	SL	INH negative	R	*katG,* wild type; *inhA* promoter, wild type	NA	*katG*, del792G (frameshift in codon 264, gcg→gc-)	Point mutation in *katG* not detected by cobas or LPA
	18001933	SL	INH negative	R	*katG*, wild type; *inhA* promoter, wild type	NA	*katG*, W397C (tgg/tgC); *mshB*, S219S (tcc/tcT); *nudC*, L293L (ctg/ctA)	Point mutations in *katG*, *mshB*, and *nudC* not detected by cobas or LPA
	17011688	SL	INH negative	R	*katG*, wild type; *inhA* promoter, wild type	NA	*katG*, del214G (frameshift in codon 72, gac→-ac); *mshB*, S219S (tcc/tcT); large unspecified deletion in *Rv3083*	Point mutations in *katG* and *mshB* not detected by cobas or LPA; deletion in *Rv3083* not detected by cobas or LPA
	17011696	SL	INH negative	R	*katG*, wild type; *inhA* promoter, wild type	NA	*katG*, M105I (atg/atA); *fabG1*, L203L (ctg/ctA)	Point mutations in *katG* and *fabG1* not detected by cobas or LPA
	17011700	SL	INH negative	R	*katG*, wild type; *inhA* promoter, wild type	NA	*katG*, M105I (atg/atA); *fabG1*, L203L (ctg/ctA); *mmaA3*, G80G (ggc/ggA)	Point mutations in *katG*, *fabG1*, and *mmaA3* not detected by cobas or LPA
	18001920	SL	INH negative	R	*katG*, wild type; *inhA* promoter, wild type	NA	*katG*, D94G (gac/gGc), N35N (aac/aaT); *Rv3083*, E123A (gag/gCg)	Point mutations in *katG* and *Rv3083* not detected by cobas or LPA
	18001921	SL	INH negative	R	*katG*, wild type; *inhA* promoter, wild type	NA	*katG*, L430P (ctg/cCg)	Point mutation in *katG* not detected by cobas or LPA
	18002310	SL	INH negative	R	*katG*, wild type; *inhA* promoter, wild type	NA	*katG*, N35N (aac/aaT), Y197D (tat/Gat); *fabG1*, L203L (ctg/ctA)	Point mutations in *katG* and *fabG1* not detected by cobas or LPA
	18002312	SL	INH negative	R	*katG*, wild type; *inhA* promoter, wild type	NA	*fabG1*, Q200Q (cag/caA); *mmaA3*, E193K (gag/Aag)	Point mutations in *fabG1* and *mmaA3* not detected by cobas or LPA
RIF	17008778	DE	RIF negative	R	*rpoB*, S531L	R	*rpoB*, S531L	No leftover primary sample for repeat testing was available. Repeat testing by cobas from culture gave a “RIF positive” result, indicating resistance.
	18000119	SL	RIF negative	R	*rpoB*, missing wild-type 4 band, probable mutation in region 516–517	R	*rpoB*, del517 (AGA)	Codon deletion in *rpoB* not identified by cobas
	18001889	DE	RIF negative	R	*rpoB*, missing wild-type 8 band, probable mutation in region 530–534	R	*rpoB*, S531F (tcg/tTC)	Uncommon amino acid exchange (S→F) in *rpoB* codon 531 not detected by cobas
	18001933	SL	RIF negative	R	*rpoB*, missing wild-type 4 band, probable mutation in region 516–517	R	*rpoB*, del518 (AAC)	Codon deletion in *rpoB* not detected by cobas
	18002314	SL	RIF negative	R	*rpoB*, missing wild-type 2/3 bands, probable mutation at codon 513	R	*rpoB*, Q513E (caa/Gaa)	Point mutation in *rpoB* not detected by cobas

a CRS, composite reference standard; DE, Germany; del, deletion; INH, isoniazid; LPA, line probe assay (HAIN MTBDR*plus*); NA, not applicable; pDST, phenotypic drug susceptibility testing; R, resistant; RIF, rifampicin; SL, Sierra Leone; WGS, whole-genome sequencing.

b As per the instruction manual of the cobas MTB-RIF/INH assay, “positive” refers to a detectable resistance-associated mutation, whereas “negative” refers to no detectable resistance-associated mutation.

c Only mutations with confirmed or likely relevance for INH or RIF resistance are presented. Uppercase letters indicate the bases that have been altered. “-” indicates a deleted base.

## DISCUSSION

Our study demonstrates that the cobas MTB assay meets the minimal performance characteristics stated in the WHO target product profile for a same-day diagnostic test detecting pulmonary TB as an alternative to smear microscopy, which is a sensitivity of >80% (smear-negative samples, >60%; smear-positive samples, 99%) and a specificity of >98% compared with culture (22). We observed that the overall sensitivity of cobas MTB (89.2%; 95% CI, 81.7 to 93.9%) was lower than in a study by Scott et al., who reported an overall sensitivity of 94.7% (95% CI, 88.1 to 98.3%) using decontaminated sputum sediments from individuals with presumptive or confirmed TB in South Africa (16). However, the proportion of smear-negative, culture-positive samples in our study was significantly larger (26.5% versus 7%) (16). In addition, Scott et al. reported a sensitivity of 81.8% (95% CI, 59.7 to 94.8%) for cobas MTB in smear-negative samples, compared to 63.0% (95% CI, 44.2 to 78.5%) in this study, which is likely a reflection of a lower bacterial burden even among smear-negative patients in Germany than among smear-negative patients in South Africa (16).

Both the sensitivity and specificity of the cobas MTB assay for detection of MTBC DNA in clinical samples were comparable to or superior to those of five other platforms mentioned in the WHO consolidated guidelines on tuberculosis (11). Therein, performance data were reported for Xpert MTB/RIF, Xpert MTB/RIF Ultra, Truenat MTB and Truenat MTB plus (Molbio Diagnostics, India), and a loop-mediated isothermal amplification assay, with overall sensitivities and specificities ranging from 73 to 90% and 96 to 98%, respectively (11). These findings indicate that the current approaches for detection of MTBC nucleic acid in clinical samples have matured to a point where significant further improvement, particularly regarding sensitivity in microscopically negative samples, will likely depend on new technological advances. The excellent overall agreement between cobas MTB and Xpert MTB/RIF found in this study further corroborates this observation (Table S1).

Overall, the cobas MTB-RIF/INH assay performed well in detecting mutations conferring resistance to INH and RIF (Table 2). For INH, all discordant results could be explained by mutations at uncommon positions within *katG* and within other genes associated with INH resistance that had not been defined as intended target mutations of the cobas MTB-RIF/INH assay (Table 3). Notably, these mutations were also missed by LPA (Table 3). For RIF, codon deletions or point mutations that had also not been included as intended target mutations explained four of the five discordant results (Table 3). All four isolates tested resistant to RIF and INH in pDST (Table 3). As a consequence, two of the corresponding patients (18001889, *katG* S315T and *rpoB* S531F; 18002314, *katG* S315T and *rpoB* Q513E [Table 3]) would have been diagnosed with presumptively INH-monoresistant TB, while the other two (18000119, *katG* del792G and *rpoB* del517; 18001933, *katG* W397C, *mshB* S219S, *nudC* L293L, and *rpoB* del518 [Table 3]) would have been diagnosed with presumptively RIF- and INH-susceptible TB based on the cobas MTB-RIF/INH results alone until pDST results became available. In contrast, employing LPA would have led to RIF resistance being inferred from missing *rpoB* wild-type bands in all four cases. These results would have likely triggered (i) DNA sequencing of *rpoB* to rule out silent mutations (which is dependent on sample quantity and equipment availability) and (ii) initiation of treatment for RIF-resistant/MDR-TB based on the assumption that any nonsilent mutation observed in the 81-bp hot spot region of *rpoB*, as well as some mutations outside this region, is clinically relevant at the current standard dose of RIF. With Xpert MTB/RIF Ultra, all four samples gave a RIF-resistant result (Table 3). As with LPA, this would have likely triggered follow-up testing and initiation of a regimen for RIF-resistant/MDR-TB.

Our findings demonstrate the geographically heterogeneous distribution of uncommon mutations conferring resistance to the most important first-line anti-TB drugs (23). In light of previous reports from Eswatini and South Africa demonstrating the spread of an MDR-MTBC outbreak strain carrying an I491F mutation in *rpoB* which remained undetected by Xpert MTB/RIF (24, 25), our observations highlight that similar events can occur anywhere and anytime. In addition, this finding underlines the potential of nationwide, WGS-based drug resistance surveys to unveil MTBC clones that escape the NAATs deployed for routine TB screening (24, 25). Recent work on genome-based prediction of drug resistance and curation of MTBC mutation databases takes into account the geographically heterogeneous prevalence of resistance-conferring mutations (26–29). However, this also has some important implications for the design and implementation of NAATs for detection of MTBC from primary samples. First, developers of novel NAATs should consider the regional pathogen diversity found in the intended target markets. Second, NAATs for prediction of RIF or INH resistance should be quickly updated if major shifts in the molecular drug resistance landscape are observed in specific regions or on a global level. Third, test performance characteristics available from the literature should be related to the regional context prior to implementation of a new NAAT for TB screening.

This study has several limitations. First, this was a retrospective study using limited volumes of stored, frozen samples from only two countries and with limited clinical metadata. Oversampling of MTBC-positive cases prevented the calculation of positive predictive values. Moreover, for Sierra Leone, only samples that grew RIF-resistant MTBC isolates were investigated, and these do not represent the large proportion of TB patients in the country who suffer from fully susceptible TB. Also, assay specificity in high-TB-burden settings will require further investigation. Lastly, the number of smear-negative, culture-positive samples available to us was limited, resulting in relatively large confidence intervals for the assay sensitivity in this sample subset. On the other hand, the fact that we were able to investigate a comparably large number of RIF- and INH-resistant samples with a broad range of mutations, and with full resolution of discordant results by WGS, represents a major strength of this study.

In conclusion, the cobas MTB and MTB-RIF/INH assays can serve as a high-throughput diagnostic platform for diagnosis of drug-sensitive or drug-resistant TB, in particular as part of population-wide screening efforts to achieve the targets outlined by the WHO End TB Strategy (4). Due to its size and throughput, the platform is particularly suited for placement in core laboratories where it could be used in an integrated diagnostic setting, including additional testing for HIV and COVID-19. The cobas MTB-RIF/INH assay could identify and differentiate both RIF- and INH-monoresistant TB from MDR-TB in the majority of the investigated samples, enabling tailored therapeutic interventions based on current guidelines and recommendations. In addition to published performance characteristics, cost, throughput, and the locally available logistic infrastructure, the choice of a NAAT for MTBC screening should be informed by genome-based regional surveillance studies to assess whether the assay will cover clones of local relevance.

## Supplementary Material

Supplemental file 1
