# Supplementary material for: Evaluation of the Roche cobas MTB and MTB-RIF/INH Assays in Samples from Germany and Sierra Leone
Source: J Clin Microbiol. 2021 Apr 20;59(5):e02983-20. doi: 10.1128/JCM.02983-20 (PMC8091830; doi:10.1128/JCM.02983-20)
Supplement: Supplemental file 1 [file JCM.02983-20-s0001.pdf]

Supplemental material

Supplemental Table 1. Agreement on detection of *Mycobacterium tuberculosis* complex DNA from primary samples between cobas MTB and Xpert MTB/RIF. Xpert MTB/RIF results were available for a subset 128 samples investigated in this study. CI, confidence interval; MTBC, *Mycobacterium tuberculosis* complex.

**cobas MTB**	**Xpert MTB/RIF**		
	**Positive**	**Negative**	**Total**
**MTBC positive**	70	0	70
**MTBC negative**	1[1]	57	58
**Total**	71	57	128
**Positive Percent Agreement (95% CI)**	98.6 (92.4, 99.9)		
**Negative Percent Agreement (95% CI)**		100.0 (93.7, 100.0)	
**Overall Percent Agreement (95% CI)**			99.2 (95.7, 99.9)

[1] The single discordant sample was smear-negative and MTBC culture-positive.

Supplemental Table 2. Frequency of rifampicin and isoniazid resistance by smear microscopy and country of sample origin. CRS, composite reference standard (phenotypic drug susceptibility testing and line probe assay); DE, Germany; INH, isoniazid; n, number of samples; RIF, rifampicin; SL, Sierra Leone.

Drug	Smear	CRS	cobas	n, DE	n, SL	n, Total
RIF	Positive	Resistant	Positive	1	35	36
			Negative	1	3	4
			Invalid	0	0	0
		Sensitive	Positive	0	1	1
			Negative	28	1	29
			Invalid	1	0	1
		**Total**		**31**	**40**	**71**
	Negative	Resistant	Positive	0	2	2
			Negative	1	0	1
			Invalid	1	0	1
		Sensitive	Positive	0	0	0
			Negative	10	1	11
			Invalid	2	0	2
		**Total**		**14**	**3**	**17**
	**Total**			**45**	**43**	**88**
INH	Positive	Resistant	Positive	3	29	32
			Negative	0	10	10
			Invalid	0	0	0
		Sensitive	Positive	0	0	0
			Negative	27	1	28
			Invalid	1	0	1
		**Total**		**31**	**40**	**71**
	Negative	Resistant	Positive	2	2	4
			Negative	1	0	1
			Invalid	0	0	0
		Sensitive	Positive	0	0	0
			Negative	9	1	10
			Invalid	2	0	2
		**Total**		**14**	**3**	**17**
	**Total**			**45**	**43**	**88**
